# Concept selection for phenotypes and diseases using learn to rank

**DOI:** 10.1186/s13326-015-0019-z

**Published:** 2015-06-01

**Authors:** Nigel Collier, Anika Oellrich, Tudor Groza

**Affiliations:** University of Cambridge, Cambridge, UK; European Bioinformatics Institute (EMBL-EBI), Cambridge, UK; Wellcome Trust Sanger Institute, Cambridge, UK; School of ITEE, the University of Queensland, St. Lucia, Australia; Garvan Institute of Medical Research, Darlinghurst, Sydney, Australia

## Abstract

**Background:**

Phenotypes form the basis for determining the existence of a disease against the given evidence. Much of this evidence though remains locked away in text – scientific articles, clinical trial reports and electronic patient records (EPR) – where authors use the full expressivity of human language to report their observations.

**Results:**

In this paper we exploit a combination of off-the-shelf tools for extracting a machine understandable representation of phenotypes and other related concepts that concern the diagnosis and treatment of diseases. These are tested against a gold standard EPR collection that has been annotated with Unified Medical Language System (UMLS) concept identifiers: the ShARE/CLEF 2013 corpus for disorder detection. We evaluate four pipelines as stand-alone systems and then attempt to optimise semantic-type based performance using several learn-to-rank (LTR) approaches – three pairwise and one listwise. We observed that whilst overall Apache cTAKES tended to outperform other stand-alone systems on a strong recall (R = 0.57), precision was low (P = 0.09) leading to low-to-moderate F1 measure (F1 = 0.16). Moreover, there is substantial variation in system performance across semantic types for disorders. For example, the concept Findings (T033) seemed to be very challenging for all systems. Combining systems within LTR improved F1 substantially (F1 = 0.24) particularly for Disease or syndrome (T047) and Anatomical abnormality (T190). Whilst recall is improved markedly, precision remains a challenge (P = 0.15, R = 0.59).

## Introduction

Phenotypes are generally regarded as the set of observable characteristics in an individual. Examples include ‘body weight loss’ and ‘abnormal sinus rhythm’. Phenotypes are important because they help to form the basis for determining the classification and treatment of a disease. Although coding systems such as the Human Phenotype Ontology (HPO) [1] and the Mammalian Phenotype Ontology (MPO) [2] have made substantial progress in organising the nomenclature of phenotypes, authors typically report their observations using the full expressivity of human language. In order to fully exploit a machine understandable representation of phenotypic findings, it is necessary to develop techniques based on natural language processing that can harmonise linguistic variation [3-5]. Furthermore, such techniques need to operate on a range of text types such as scientific articles, clinical trials and patient records [6] in order to enable applications that require inter-operable semantics. Use cases might include automated cohort extraction to support research into a particular rare genetic disorder or support for curating databases of human genetic diseases such as the Online Mendelian Inheritance in Man database (OMIM) [7]. We envision the final result to be a representation that decomposes the phenotype terms according to their elementary conceptual units (‘building block concepts’) and harmonises them to ontologies such as the Foundational Model of Anatomy (FMA) [8] for anatomical structures, the Phenotype Attribute and Trait Ontology (PATO) [9] for qualities and Gene Ontology (GO) [10] for biological processes. Our view is that the techniques must be able to support the capture of phenotypes from both physical objects and processes as well as cutting across levels of granularity from the molecular level to the organism level.

Finding the names of technical terms in life science texts – known as named entity recognition – has been the topic of intensive study over the last decade. Grounding or normalising these terms to a logically structured domain vocabulary – an ontology – has proven to be a substantial challenge, e.g. [11,12], because of idiosyncrasies in naming, the need to exploit syntactic structure in the case of disjoint terms, the paucity of annotated corpora for training and evaluation and the incompleteness of the target ontologies themselves. To accomplish this task, concept identification systems have emerged with different analytical goals. In this paper, we investigate the utility of four existing conceptual coding pipelines (i.e. MetaMap [13], Apache cTAKES [14], NCBO annotator [15] and BeCAS [16]) in order to identify and harmonise the phenotypes and other concepts related to the diagnosis and treatment of diseases. These tools do not explicitly consider phenotypes as a conceptual category but rather provide groundings from text to a range of building block concepts which we hope to exploit. In order to provide a basis for comparing these tools quantitatively and qualitatively, we have chosen to harmonise their outputs to Unified Medical Language System (UMLS) concept unique identifiers (CUIs) and semantic types as the common coding standard. Concept unique identifiers provide a way to encode senses of words and phrases, e.g. *culture* as either ‘anthropological culture’ or ‘laboratory culture’ [17]. UMLS semantic types provide a broad classification of all the UMLS concepts contained in the MetaThesaurus as well as a structuring of those semantic types. There are approximately 133 semantic types and 54 relationships between them. UMLS annotations were assigned at the sentence level. Textual annotations used the ShARE/CLEF 2013 corpus [18] which we describe later. We have identified the concept classes which are the most promising building blocks – such as T184 Sign and Symptom - and evaluated based on these. Our approach aims to work towards the composition of phenotypes in future work based on the building block outputs of the systems reported here. We chose to focus on the uncustomised use-case of the four base systems as a way of exploring their immediate utility to users who did not have access or resources to build annotated training data or the ability to build their own post-processing rules.

In addition to evaluating the suitability of each individual system on the ShARE/CLEF 2013 corpus, we investigate possibilities to optimise the outputs of systems using an ensemble approach. In order to take advantage of the complementarity in concept recognition and go beyond a simple voting mechanism, we have employed several learn-to-rank (LTR) methods – more specifically three pairwise ranking approaches: SVMRank [19], RankBoost [20] and RankNet [21]; and one listwise ranking approach: ListNet [22]. Such methods learn to optimise contraints pairwise or list wise based on a set of features and a predefined ranking of the input. In our setting, each sentence, treated as an instance, is described via five feature blocks by the individual CR systems. Using the ShARE/CLEF 2013 training data, the ranking of the systems is assigned based on the ground truth and a model is learned such that it maximises the ranking correlation. The final optimised ensemble and model is tested on the ShARE/CLEF 2013 test data set. The learn to rank ensemble enables us to accept the choices of more than one system in the event of a closely tied ranking. We found that combining systems within learn to rank improved F1 substantially compared to stand-alone systems.

## Methods

### Data

For evaluation and training the re-ranker we chose to use the ShARE/CLEF e-health 2013 Task 1 evaluation data set of 300 de-identified clinical records from the Multiparameter Intelligent Monitoring in Intensive Care (MIMIC) II data-base (http://mimic.physionet.org/database.html) with stand-off annotations for disorders. This is a mixed corpus that includes discharge summaries, echo reports and radiology reports used in an intensive care unit setting. 200 notes were designated for training and 100 for testing. Annotation was done by two annotators plus open adjudication. Access to the corpus required appropriate registration with MIMIC II and the completion of a US human subjects training certificate. The distribution of UMLS semantic types for disorder-related text spans can be seen in Tables 1 and 2. Note that we removed minor classes with frequencies of 1 (i.e. T002, T031, T049, T058, T059, T121, T197). As can be seen in Tables 1 and 2, the majority of semantic types relate to diseases, symptoms and pathological functions together with a substantial minority of annotations for injuries, congenital and anatomical abnormalities and mental/behavioral dysfunctions.

**Table 1:** **ShARE/CLEF e-health training corpus semantic types**

**ID**	**UMLS Semantic type**	**Freq.**	**Unique**	**Av. term length**
T047	Disease or syndrome	1803	410	1.97
T184	Sign or symptom	842	163	1.56
T046	Pathologic function	518	133	1.65
T037	Injury or poisoning	213	96	2.00
T019	Congenital abnormality	184	25	3.61
T190	Anatomical abnormality	103	36	1.77
T191	Neoplastic process	92	49	1.87
T048	Mental or behavioral dysfunction	84	32	1.76
T033	Finding	45	15	2.90
T020	Acquired abnormality	40	17	1.93

Distribution of UMLS semantic types for annotations by frequency and frequency without duplication as well as the average term length in tokens.

**Table 2:** **ShARE/CLEF e-health test corpus semantic types**

**ID**	**UMLS Semantic type**	**Freq.**	**Unique**	**Av. term length**
T047	Disease or syndrome	1723	371	1.88
T184	Sign or symptom	816	149	1.51
T046	Pathologic function	520	113	1.59
T037	Injury or poisoning	106	33	1.75
T019	Congenital abnormality	96	18	1.88
T190	Anatomical abnormality	125	26	1.74
T191	Neoplastic process	73	34	2.02
T048	Mental or behavioral dysfunction	137	32	1.67
T033	Finding	13	6	1.11
T020	Acquired abnormality	41	21	1.62

Distribution of UMLS semantic types for annotations by frequency and frequency without duplication as well as the average term length in tokens.

**Figure 1:**
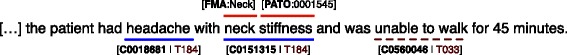
Example of sentence annotations from the ShARE/CLEF corpus. The example shows concept annotations for ‘headache; (C0018681 | T184), ‘neck stiffness’ (CO151315 | T184) and ‘unable to walk’ (C0560048 | T033). An example decomposition for ‘neck stiffness’ is shown with an illustrative mapping to PATO:0001545 (‘inflexible’) and FMA:Neck.

An example source sentence from the corpus is shown in Figure 1 along with actual gold standard concept annotations, harmonized semantic types and a potential decompositional mapping to PATO and FMA for one clinical phenotype (‘neck stiffness’). Here “C*” annotations correspond to “concept annotations” and “T*” annotations correspond to “harmonised semantic types”.

The distributions for train and test possess good agreement but, at the same time, also interesting differences: the average length of mentions of T019 *congenital abnormality* appears remarkably longer in the training corpus, and there are relatively fewer T037 *injury or poisoning* and T019 *congenital abnormality* instances in the testing set. Moreover, we observe a greater variety of T037 instances in the testing corpus.

Examples of what we might consider interesting phenotypes occur across all anntoated UMLS semantic types as well as for unannotated strings. For example, ‘Right ventricular [is mildly] dilated’ (C0344893 | T019), ‘wall motion abnormality’ (no CUI) and ‘hypotension’ (C0520541 | T047). In other cases, the class shows a disease and not a phenotype, e.g. ‘complex autonomous disease’ (C0264956 | T046). We note that unannotated strings were not explicitly quantified in the present study reported here and and are left for future study.

### Experimental setup

We follow standard metrics of evaluation for the task using F1, i.e. the harmonic mean of recall (R) and precision (P). This is the same metric used by participants of the ShARE/CLEF 2013 Task 1. F1 is calculated as F1 = 2PR/(P + R), with P = TP/(TP + FP) and R = TP/(TP + FN) where TP is the number of system suggestions where the semantic type and the CUI is the same as the gold standard; FP is the number of system suggestions where the semantic type and/or the CUI do not match the gold standard; and FN is the number of spans in the gold standard which the system failed to suggest. The major difference between our evaluation and the ShARE/CLEF shared task is that we evaluate at the sentence level and not the mention level, i.e. the focus is on predicting concept labels for the sentence as a whole and not the starting and ending positions of those annotations in the sentence. Consequently, our experimental results are not directly comparable with those achieved by systems participating in the ShARE/CLEF Tasks. Evaluation is conducted using blind data not used in system development data or training.

Different applications require a different approach to defining a true positive, false negative etc. In this case we have considered a correct match to be recorded when a complete match occurs between system output and gold standard for both the identifier and the semantic type of that concept in UMLS. In line annotation is not considered explicitly within this evaluation. Clearly any further application requiring the explicit annotation of relationships between concepts within the sentence would require this. The evaluation protocol reported here supports use cases such as statistical association analysis between the co-occurring concepts and document indexing/retrieval.

### Individual system descriptions

The problem we consider is how to select a set of disorder-related SNOMED CT concepts for any given sentence. Disorder-related concepts are chosen because of their relevance to phenotype recognition. SNOMED-CT was chosen as the ontology for harmonisation because it offers a joint coding ontology for all the base systems. A number of factors complicate the task including: (a) in line with our desire to test off-the-shelf performance, the system pipelines were not tuned in any way for predicting the specific set of disorder-related semantic types appearing in the corpus, (b) the annotation scheme allows for disjoint (e.g. ‘Right ventricular … dilated’) and overlapping annotation spans; and (c) clinical texts contain a high number of abbreviations causing additional complications for term identification and harmonisation.

We consider four uncustomized base concept annotation systems based on clinical natural language processing: NCBO Annotator, BeCAS, cTAKES and MetaMap. With the exception of MetaMap, all the other systems were used with their default parameters. Other systems that could have been applied here include ConceptMapper [23], Whatizit [24] and Bio/MedLee [25]. These systems were either difficult to access or did not provide a route to UMLS concept harmonizations. The systems we applied adopt a range of techniques but tend to avoid deep parsing. Instead, they make use of a range of shallow parsing, sequence-based machine learning (e.g. for named entity recognition and part of speech tagging) and pattern-based techniques, supplemented with restrictions and inferences on source ontologies such as SNOMED CT [26]. In all cases it should be noted that we dealt with black box systems.

**NCBO Annotator (M1)** The NCBO Annotator is an online system that identifies and indexes biomedical concepts in unstructured text by exploiting a range of over 300 ontologies in BioPortal. These ontologies include many that have particular relevance to disorders and phenotypes such as SNOMED CT, LOINC (Logic Observation Identifiers, Names and Codes) [27], the FMA and the International Classification of Diseases (ICD-10) [28]. NCBO Annotator operates in two stages: concept recognition and semantic expansion. Concept recognition performs lexical matching by pooling terms and their synonyms from across the ontologies and then applying a multiline version of grep to match lexical variants in free text. During semantic expansion, various rules such as transitive closure and semantic mapping using the UMLS Metathesaurus are used to suggest related concepts from within and across ontologies based on extant relationships.

**BeCAS (M2)** BeCAS (the BioMedical Concept Annotation System) is the newest integrated system of the four that we tried. The pipeline of processes involves the following stages: sentence boundary detection, tokenization, lemmatization, part of speech (POS) tagging and chunking, abbreviation disambiguation, and concept unique identifier (CUI) tagging. The first four stages are performed by a dependency parser that incorporates domain adaptation using unlabelled data from the target domain. CUI tagging is conducted using regular expressions for specific types such as anatomical entities and diseases. Dictionaries used as sources for the regular expressions include the UMLS, LexEBI [29] and the Jochem joint chemical dictionary [30]. During development the concept recognition system was tested on abstracts and full length scientific articles using an overlapping matching strategy.

**Apache cTAKES (M3)** cTAKES consists of a staged pipeline of modules that are both statistical and rule-based. The order of processing is somewhat similar to MetaMap and consists of the following stages: sentence boundary detection with OpenNLP, tokenization, lexical normalisation (SPECIALIST lexical tools), part of speech tagging and shallow parsing using OpenNLP trained in-domain on Mayo Clinic EPR concept recognition, negation detection using NegEx [31] and temporal status detection. Concept recognition is conducted within the boundaries of noun phrases using dictionary matching on a synonym-extended version of SNOMED CT and RxNORM [32] subset of UMLS. Evaluation was conducted with a focus on EPRs but also using corpora from the scientific literature.

**MetaMap (M4-M9)** MetaMap is a widely used and technically mature system from the National Library of Medicine (NLM) for finding mentions of clinical terms based on CUI mappings to the UMLS Metathesaurus. The UMLS Metathesaurus forms the core of the UMLS and incorporates over 100 source vocabularies including the NCBI taxonomy, SNOMED CT and OMIM. Output is to the 135 UMLS semantic types. The system exploits a fusion of linguistic and statistical methods in a staged analysis pipeline. The first stages of processing perform mundane but important tasks such as sentence boundary detection, tokenization, acronym/abbreviation identification and POS tagging. In the next stages, candidate phrases are identified by dictionary lookup in the SPECIALIST lexicon and shallow parsing using the SPECIALIST parser. String matching then takes place on the UMLS Metathesaurus before candidates are mapped to the UMLS and compared for the amount of variation. A final stage of word sense disambiguation uses local, contextual and domain-sensitive clues to arrive at the correct CUI.

MetaMap is unique in providing a rich set of options [33] to allow the user to customise the approach the system takes to concept mapping. We chose to explore a range of options including what we considered a high precision ’strict’ approach to matching as well as negation detection with NegEx. The variations of MetaMap we explored were:

- M4: MetaMap -A -negex — using strict matching and negation detection
- M5: MetaMap -A -y — using strict matching and forcing MetaMap to perform word sense disambiguation on equally scoring concepts
- M6: MetaMap -g — allowing concept gaps
- M7: MetaMap -i — ignoring word order
- M8: MetaMap — using the base version
- M9: MetaMap -A — using strict matching only

### Ensemble approach

In addition to the nine basic systems M1 to M9, we evaluated several ranking approaches that rank the quality of basic system outputs based on a sentence-level and concept-level features. These features include individual source sentence vocabulary, the semantic types suggested by the system and the vocabulary for the suggested concept labels. More sophisticated features will be tested in the future. We believe that the chosen features serve as a useful first step for evaluating the ranking approach. The approaches we tested make use of a scoring function to rank each system’s output set of concept labels against the training data. These rankings are used together with the features to train a learn-to-rank (LTR) model. We evaluated four different ranking algorithms based on pairwise and list wise comparisons to maximise the ranking correlation for all categories, where the categories represent the nine basic systems. We explore the underlying assumption that a set of features exists that can predict when one system will perform better on a given sentence than another. The ranking function we applied was the F1 metric that we used to evaluate each system described in detail in the section below.

Ranking essentially aims to establish which hypothesis about sentence-level concept annotations is most likely given the available evidence. Labelled instances are provided during training as feature vectors. Each label denotes a single rank that is determined by comparing the F1 scores for each system based on the concepts they output on that sentence against the set of gold standard concepts. The goal of training each of the ranking approaches is to find a model that correctly determines the ordering of systems on a given sentence. Afterwards we can either choose the predictions from the single highest ranking system or combine a group of highly ranking systems.

The feature blocks used by the ensemble model are listed in Table 3. During testing, a feature vector is provided for each system (methods M1 … M9) and the LTR model determines a score which is then converted to an ordered ranked list by the ensemble. In practice the semantic types suggested by the top system are selected. If the first rank is shared between multiple systems, the top outputs from the top ranking systems are combined by taking the union.

**Table 3:** **Feature blocks used to build the ensemble model**

**Feature block**	**Description**
FB1	A Boolean set of features for the system identifiers (i.e. M1 … M9);
FB2	A Boolean set of features for the semantic types that are predicted by the system to appear and not appear in the sentence (i.e. T047, T184, … etc.);
FB3	A set of integer valued features for the counts of vocabulary terms appearing in UMLS concepts that are predicted by the systems to appear in the sentence; In total the set consisted of 1,008 UMLS CUIs;
FB4	A set of integer valued features for the counts of vocabulary terms appearing in the sentence; The vocabulary consisted of 13,565 terms;
FB5	A set of integer valued features for the ‘45 cluster’ distributed semantic classes which match to FB3. The 45 cluster classes derived by Richard Socher and Christoph Manning from PubMed are available at http://nlp. stanford.edu/software/ bionlp2011-distsim-clusters-v1.tar.gz

The LTR systems that we investigated include three pairwise LTR – SVMRank [19], RankNet [21], and RankBoost [20] – and one listwise LTR – ListNet [22]. Table 4 provides a succinct comparative overview of the two types of LTR, as initially described in [34].

**Table 4:** **Brief comparative overview on the learn to rank approaches, adapted from [** 34 **]**

	**Pairwise learn to rank**	**Listwise learn to rank**
**Goal**	Ranking by learning on object pairs	Ranking by learning on object lists
**Loss function**	pairwise loss, e.g., hinge loss, exponential loss, logistic loss	listwise loss, e.g., cross entropy loss, cosine loss
**Advantages**	Theoretical aspects are well studied	Considers the relationship among objects to their full extent
**Disadvantages**	Considers only pairwise orders; May be biased towards lists with more objects	Theoretical aspects are less well studied
**Algorithms**	SVMRank [19]; RankNet [21]; RankBoost [20]	ListNet [22]

## Results

### Comparison of stand-alone systems

Table 5 presents results for each of the stand-alone systems at a macro level, while Table 6 lists results structured according to semantic type. Note that we did not perform any learning procedure at this stage on the gold standard corpus. We can see several noteable results including the relatively better performance of system M3 (cTAKES), both at the macro level (0.16 F1, compared to 0.08 F1 achieve by the next system in line – M5), as well as across most semantic types – with the exception of T190 (Anatomical abnormality) where system M4 does best. No single system though achieves both winning recall and precision in the type-based setting. System M5 for example (MetaMap -A -y) generally achieves the highest precision. We can also note a wide disparity in F1 by systems across semantic types.

**Table 5:** **Comparison of stand-alone systems on training data**

**System**	**P**	**R**	**F1**
M1: NCBO Annotator	0.0393	0.5044	0.0729
M2: BeCAS	0.0146	0.0134	0.0140
M3: Apache cTAKES	0.0933	0.5675	**0.1602**
M4: MetaMap -A -negex	0.0389	0.2992	0.0689
M5: MetaMap -A -y	0.0498	0.2505	0.0831
M6: MetaMap -g	0.0387	0.2905	0.0683
M7: MetaMap -i	0.0392	0.2994	0.0693
M8: MetaMap	0.0389	0.2992	0.0689
M9: MetaMap -A	0.0389	0.2992	0.0689

Macro precision, recall and F1 of the individual systems on the training data. The highest scoring system F1 is shown in bold.

**Table 6:** **Comparison of stand-alone systems on training data**

**ID**	**Sys**	**P**	**R**	**F1**	**ID**	**Sys**	**P**	**R**	**F1**
T047	M1	0.39	0.55	0.45	T191	M1	0.24	0.30	0.26
	M2	0.03	0.01	0.02		M2	0.05	0.03	0.04
	M3	0.44	0.63	**0.52**		M3	0.29	0.64	**0.40**
	M4	0.58	0.28	0.38		M4	0.21	0.25	0.23
	M5	*0.72*	0.22	0.34		M5	*0.38*	0.23	0.28
	M6	0.58	0.27	0.37		M6	0.21	0.25	0.23
	M7	0.58	0.28	0.38		M7	0.22	0.25	0.23
	M8	0.58	0.28	0.38		M8	0.21	0.25	0.23
	M9	0.58	0.28	0.38		M9	0.21	0.25	0.23
T184	M1	0.35	0.61	0.45	T048	M1	0.28	0.49	0.35
	M2	0.02	0.01	0.01		M2	0.04	0.03	0.03
	M3	0.47	0.58	**0.52**		M3	0.45	0.55	**0.50**
	M4	0.62	0.41	0.49		M4	0.53	0.34	0.42
	M5	*0.68*	0.36	0.47		M5	*0.67*	0.27	0.38
	M6	0.61	0.40	0.49		M6	0.54	0.35	0.43
	M7	0.61	0.41	0.49		M7	0.54	0.34	0.42
	M8	0.62	0.41	0.49		M8	0.53	0.34	0.42
	M9	0.62	0.41	0.49		M9	0.53	0.34	0.42
T046	M1	0.28	0.62	0.39	T033	M1	0.01	0.36	0.01
	M2	0.03	0.04	0.03		M2	0.00	0.00	0.00
	M3	0.30	0.69	**0.42**		M3	0.00	0.11	0.00
	M4	0.50	0.34	0.40		M4	0.00	0.13	0.01
	M5	*0.50*	0.26	0.34		M5	0.00	0.13	0.01
	M6	0.49	0.33	0.39		M6	0.00	0.13	0.01
	M7	0.50	0.34	0.41		M7	0.00	0.13	0.01
	M8	0.50	0.34	0.40		M8	0.00	0.13	0.01
	M9	0.50	0.34	0.40		M9	0.00	0.13	0.01
T037	M1	0.19	0.24	0.21	T020	M1	0.36	0.50	**0.42**
	M2	0.00	0.00	0.00		M2	0.00	0.00	0.00
	M3	0.26	0.34	**0.30**		M3	0.33	0.57	**0.42**
	M4	0.38	0.22	0.28		M4	0.36	0.36	0.36
	M5	*0.42*	0.21	0.28		M5	*0.36*	0.21	0.27
	M6	0.36	0.20	0.25		M6	0.36	0.33	0.35
	M7	0.37	0.21	0.27		M7	0.36	0.36	0.36
	M8	0.38	0.22	0.28		M8	0.36	0.36	0.36
	M9	0.38	0.22	0.28		M9	0.36	0.36	0.36
T190	M1	0.12	0.44	0.19	T019	M1	0.40	0.11	0.18
	M2	0.01	0.01	0.01		M2	0.00	0.00	0.00
	M3	0.12	0.55	0.19		M3	0.58	0.14	**0.23**
	M4	0.28	0.22	**0.25**		M4	0.27	0.07	0.11
	M5	*0.32*	0.19	0.24		M5	0.34	0.06	0.11
	M6	0.28	0.22	**0.25**		M6	0.25	0.07	0.11
	M7	0.28	0.22	**0.25**		M7	0.27	0.07	0.11
	M8	0.28	0.22	**0.25**		M8	0.27	0.07	0.11
	M9	0.28	0.22	**0.25**		M9	0.27	0.07	0.11

Type-based micro precision, recall and F1 of the individual systems on the training data. The highest scoring system F1 for each semantic type is shown in bold. Note that italics scores indicate the highest level achieved for recall and precision for each semantic type by any system.

In general the stand-alone systems performed better on T047, T184 and T048. In contrast, performance on T037, T190, T033 and T019 tended to be weak. Stronger performance might be partly correlated with shorter average term length (see Table 2) but this is not an entirely satisfying explanation. Another possible explanation is hinted at by the fact that the more challenging classes are at the lower end of frequencies in the EPR data. This might indicate that the semantic resources which the systems draw on have been less intensively developed and might not provide such extensive lexical support as more frequent classes.

### Learn-to-rank results

Using documents as the sampling unit, we performed randomised 10-fold cross validation on the ShARE/CLEF training data. 9 parts of the data were selected without replacement to train the four LTR models from scratch and 1 part was used to test. The 10 test parts were then joined together and recall, precision and F-score were calculated as in the stand-alone evaluation.

In the testing stage, we experimented with all combinations of feature blocks and also with different settings for LTR parameters. Best results were achieved using feature blocks FB1, FB2 and FB4, in addition to the following model parameters:

- SVMRank: a value of 30 for the trade-off between training error and margin;
- RankNet: 100 epochs, 1 hidden layer with 10 nodes and a learning rate of 0.00005;
- RankBoost: 300 rounds to train and 10 threshold candidates to search;
- ListNet: 1500 epochs and a learning rate of 0.00001;

Feature blocks FB3 and FB5 were not found to improve performance in these experiments.

Finally, in order to gain a deeper understanding in the ensembles’ behaviour, we have experimented with different tie-breaking strategies, at different top-K ranking levels. In our context, the outcome of applying LTR on an instance is a ranked list of the individual systems for that instance, together with the associated weight. Hence, to compute the standard performance metrics such that the results are comparable to those of the stand-alone systems, the LTR ranking has to be transformed into a hard classification outcome. This is realised by introducing a cut-off at a desired top-K level, which entails that the systems ranked above K will participate in the classification outcome. In a setting where multiple systems may be ranked above the threshold (possible even for K = 1), a tie-breaking strategy is required. We have considered two strategies: (i) a union strategy, where the individual annotations of all top-K ranked systems are merged via a set union, and the union is considered the final classification result on that particular instance; and (ii) an oracle strategy, where using the ground truth, we aim to choose the single system among the top-K ranked that maximises the performance metric.

While the first strategy does not require any a priori knowledge and is usable in a proper application scenario, the second is only usable when the ground truth is known. Thus, it is not applicable for appropriate testing. However, we included this strategy in order to understand the actual contribution of the individual systems to the ensemble result. Consequently, the tables listing the macro-performance metrics of the ensemble on both the ten-fold cross validation (Table 7 and 8), as well as on the blind test data (Table 9) are accompanied by a measure of individual system contribution to the final outcome. Note that, under normal circumstances, the sum of all individual contributions should be 1.0. However, this is true only for the oracle strategy, where a single system is chosen to represent the ensemble. The union strategy may involve several systems, each of which will score points for contributing to the ensemble result.

**Table 7:** **Learn to rank on training data**

**Learn to rank performance**						**Individual system contribution**								
**Top-K**	**Strategy**	**Model**	**P**	**R**	**F1**	**M1**	**M2**	**M3**	**M4**	**M5**	**M6**	**M7**	**M8**	**M9**
Top-1	Union	SVMRank	0.1513	0.5960	**0.2413**	0.25	0.04	*0.45*	0.01	0.00	0.01	0.00	-	0.23
		ListNet	0.1153	0.5880	0.1928	*0.73*	0.03	0.21	0.02	-	0.00	-	-	-
		RankNet	0.0924	0.5206	0.1570	*1.00*	-	-	-	-	-	-	-	-
		RankBoost	0.1296	0.6125	0.2139	0.46	0.05	*0.50*	-	-	0.01	0.28	0.28	0.28
	Oracle	SVMRank	0.1513	0.5960	0.2413	0.25	0.04	*0.45*	0.01	0.00	0.01	0.00	-	0.23
		ListNet	0.1153	0.5880	0.1928	*0.73*	0.03	0.21	0.02		0.00	-	-	-
		RankNet	0.0924	0.5206	0.1570	*1.00*	-	-	-	-	-	-	-	-
		RankBoost	0.1791	0.6113	**0.2770**	0.27	0.04	*0.49*	-	-	0.01	0.18	0.00	-
Top-2	Union	SVMRank	0.1122	0.6426	**0.1911**	0.58	0.07	*0.66*	0.03	0.00	0.02	0.00	0.23	0.40
		ListNet	0.0996	0.6566	0.1730	*0.94*	0.06	0.88	0.10	0.01	0.01	0.00	-	-
		RankNet	0.0989	0.6477	0.1716	*1.00*	-	*1.00*	-	-	-	-	-	
		RankBoost	0.1084	0.6469	0.1857	0.55	0.09	0.61	0.01	0.00	0.28	*0.76*	*0.76*	-
	Oracle	SVMRank	0.2340	0.6316	0.3415	0.09	0.00	*0.67*	0.01	0.00	0.01	-	-	0.23
		ListNet	0.2390	0.6439	0.3486	0.19	0.00	*0.75*	0.05	0.01	0.00	-	-	-
		RankNet	0.2533	0.6363	**0.3624**	0.17	-	*0.83*	-	-	-	-	-	-
		RankBoost	0.2385	0.6359	0.3469	0.11	0.00	*0.57*	-	-	0.02	0.29	0.00	-
Top-3	Union	SVMRank	0.1051	0.6545	**0.1811**	0.61	0.18	*0.68*	0.07	0.02	0.03	0.24	0.40	0.77
		ListNet	0.0921	0.6761	0.1621	*0.97*	0.60	0.96	0.40	0.03	0.03	0.01	-	-
		RankNet	0.0943	0.6486	0.1647	*1.00*	*1.00*	*1.00*	-	-	-	-	-	
		RankBoost	0.1048	0.6532	0.1806	0.56	0.11	0.63	0.29	0.23	0.75	*0.97*	*0.97*	-
	Oracle	SVMRank	0.2469	0.6409	0.3565	0.07	0.00	*0.62*	0.02	0.01	0.01	0.00	-	0.28
		ListNet	0.2716	0.6596	**0.3848**	0.13	0.00	*0.72*	0.14	0.01	0.00	-	-	-
		RankNet	0.2536	0.6367	0.3627	0.17	0.00	*0.83*	-	-	-	-	-	-
		RankBoost	0.2553	0.6397	0.3650	0.10	0.00	*0.56*	-	0.09	0.02	0.23	0.00	-

Macro precision, recall and F1 at different top K levels. The highest scoring system F1 for each level (both union and oracle strategies) is shown in bold. The table also shows the individual contribution of the systems to the final score where italics scores indicate the highest contributing individual system(s) to each ensemble.

**Table 8:** **Type-based learn to rank on training data**

**ID**	**Sys**	**P**	**R**	**F1**	**ID**	**Sys**	**P**	**R**	**F1**
T019	SVMRank	0.46	0.15	**0.23**	T047	SVMRank	0.53	0.64	**0.58**
	ListNet	0.47	0.13	0.20		ListNet	0.42	0.65	0.51
	RankNet	0.47	0.11	0.18		RankNet	0.43	0.57	0.49
	RankBoost	0.43	0.16	**0.23**		RankBoost	0.46	0.68	0.55
T020	SVMRank	0.32	0.57	**0.41**	T048	SVMRank	0.44	0.62	0.52
	ListNet	0.29	0.50	0.36		ListNet	0.42	0.54	0.47
	RankNet	0.33	0.42	0.37		RankNet	0.39	0.51	0.44
	RankBoost	0.21	0.50	0.30		RankBoost	0.48	0.67	**0.56**
T033	SVMRank	0.01	0.32	0.02	T184	SVMRank	0.46	0.63	**0.53**
	ListNet	0.01	0.48	0.02		ListNet	0.40	0.66	0.50
	RankNet	0.01	0.48	**0.03**		RankNet	0.39	0.62	0.48
	RankBoost	0.01	0.45	0.02		RankBoost	0.45	0.64	**0.53**
T037	SVMRank	0.34	0.33	**0.33**	T190	SVMRank	0.20	0.61	**0.30**
	ListNet	0.26	0.24	0.25		ListNet	0.17	0.58	0.27
	RankNet	0.22	0.21	0.22		RankNet	0.15	0.44	0.22
	RankBoost	0.30	0.29	0.29		RankBoost	0.16	0.60	0.25
T046	SVMRank	0.29	0.66	**0.40**	T191	SVMRank	0.31	0.56	0.40
	ListNet	0.25	0.67	0.36		ListNet	0.37	0.44	0.40
	RankNet	0.27	0.61	0.37		RankNet	0.35	0.36	0.36
	RankBoost	0.24	0.67	0.35		RankBoost	0.35	0.56	**0.43**

Note that the highest scoring system F1 for each semantic type is shown in bold.

**Table 9:** **Learn to rank on test data**

**Learn to rank performance**						**Individual system contribution**								
**Top-K**	**Strategy**	**Model**	**P**	**R**	**F1**	**M1**	**M2**	**M3**	**M4**	**M5**	**M6**	**M7**	**M8**	**M9**
Top-1	Union	SVMRank	0.1712	0.6426	**0.2703**	0.17	0.04	*0.50*	-	-	0.01	0.00	-	0.28
		ListNet	0.1271	0.6170	0.2108	*0.65*	0.04	0.26	0.05	-	0.00	-	-	-
		RankNet	0.0923	0.5096	0.1562	*1.00*	-	-	-	-	-	-	-	-
		RankBoost	0.1408	0.6524	0.2316	0.43	0.05	*0.50*	-	-	0.01	0.28	0.28	0.28
	Oracle	SVMRank	0.1712	0.6426	0.2703	0.17	0.04	*0.50*	-	-	0.01	0.00	-	0.28
		ListNet	0.1271	0.6170	0.2108	*0.65*	0.04	0.26	0.05	-	0.00	-	-	-
		RankNet	0.0923	0.5096	0.1562	*1.00*	-	-	-	-	-	-	-	-
		RankBoost	0.1872	0.6504	**0.2907**	0.23	0.05	*0.51*	-	-	0.01	0.20	0.00	-
Top-2	Union	SVMRank	0.1244	0.6986	**0.2112**	0.51	0.07	*0.62*	-	-	0.01	0.00	0.28	0.50
		ListNet	0.1107	0.7109	0.1915	0.87	0.07	*0.88*	0.13	0.03	0.02	0.00	-	-
		RankNet	0.1070	0.7028	0.1857	*1.00*	-	*1.00*	-	-	-	-	-	-
		RankBoost	0.1188	0.7034	0.2032	0.53	0.09	0.62	0.01	0.01	0.28	*0.76*	*0.76*	*0.76*
	Oracle	SVMRank	0.2350	0.6869	0.3501	0.07	0.00	*0.63*	-	-	0.00	0.00	-	0.29
		ListNet	0.2534	0.6981	0.3718	0.14	0.00	*0.77*	0.06	0.02	0.00	0.00	-	-
		RankNet	0.2629	0.6905	**0.3808**	0.15	-	*0.85*	-	-	-	-	-	-
		RankBoost	0.2420	0.6908	0.3584	0.10	0.00	*0.58*	-	0.01	0.02	0.29	0.01	-
Top-3	Union	SVMRank	0.1157	0.7081	**0.1990**	0.53	0.10	0.63	-	0.01	0.28		0.50	*0.93*
		ListNet	0.1019	0.7287	0.1788	0.91	0.57	*0.92*	0.44	0.09	0.06	0.02	0.00	-
		RankNet	0.1029	0.7045	0.1796	*1.00*	*1.00*	*1.00*	-	-	-	-	-	-
		RankBoost	0.1128	0.7109	0.1947	0.54	0.11	0.64	0.29	0.22	0.75	*0.98*	*0.98*	*0.98*
	Oracle	SVMRank	0.2444	0.6933	0.3615	0.06	0.00	*0.59*	-		0.01	0.00	-	0.33
		ListNet	0.2773	0.7126	**0.3993**	0.11	0.00	*0.72*	0.12	0.04	0.01	0.00	-	-
		RankNet	0.2643	0.6914	0.3824	0.15	0.01	*0.85*	-	-	-	-	-	-
		RankBoost	0.2593	0.6956	0.3777	0.09	0.00	*0.57*	-	0.10	0.02	0.22	0.00	-

Macro precision, recall and F1 at different top K levels. The highest scoring system F1 for each level (both union and oracle strategies) is shown in bold. The table also shows the individual contribution of the systems to the final score where italics scores indicate the highest contributing individual system(s) to each ensemble.

Returning to the results, the overall macro performance of the LTR approaches on ten-fold cross validation using the ShARE/CLEF training set is listed in Table 7. At top-1 rank level, the best union strategy was achieved by SVMRank with an F1 of 0.24, while the Oracle strategy shows RankBoost to outperform the other models with an F1 of 0.28. These compare to the best single system, as shown in Table 5, which was cTAKES (M3) with F1 = 0.16 – representing a contribution of +8 and +11 points of F1, respectively. The decrease in ranking threshold leads to a natural decrease in F-Score for the union strategy (since it become more and more inclusive), and at the same time with an increase in F-Score for the Oracle strategy (since it enlarges the pool from which it can choose the optimal solution) – from 0.24 (top-1) to 0.19 (top-2) and 0.18 (top-3) for union and from 0.28 (top-1) to 0.36 (top-2) and 0.38 (top-3) for Oracle. Independently of the threshold or model, however, the results of the stand-alone systems are reflected in the individual contributions of the systems in the ensemble (as shown in Table 7). With a few exceptions, most of which are in the union strategy, M3 (cTakes) is the most prominent contributor to the ensemble outcome, paired, subject to the LTR model, either with M1 (NCBO Annotator) or M9 (MetaMap strict).

It is interesting to note that, while using the Union strategy the LTR outcome is consistent across different top-K levels – SVMRank achieving the best results – the same does not hold for the Oracle strategy, which shows three models achieving the best results at three top-K levels. There are, however, some patterns that emerge from the individual system contributions. For example, RankNet shows a clear preference towards M1 and M3 only. SVMRank, ListNet and RankBoost use predominantly M1 and M3, augmented with M9, M4 and M7 respectively. Surprisingly M4 (MetaMap with Negex) appeared to have minimal impact in the ensemble although it features more prominently in several Oracle experiments.

The ensemble approach improved performance for all semantic types with the exception of two cases, where the performance was slightly reduced: T046 (F1: 0.42 to 0.40), T020 (F1: 0.42 to 0.41). More importantly, in some cases, the improvement was substantial, e.g., 6% on T047 and T048 or 5% on T190. In terms of LTR model, different models preferred different types – the results being split between SVMRank and RankBoost. T047, T184 and T190 were dominated by SVMRank and T191 and T084 by RankBoost.

In order to show the generalizability of the ensembles, we ran them on the ShARE/CLEF held out set. The overall results listed in Table 9 show an average improvement in performance of 2% across different tie-breaking strategies and top-K levels. Furthermore, the individual system contributions follow the same patterns as discussed on the cross-validation results. Finally, as shown in Table 10, most semantic types achieved stronger performance on the testing data with T019, T037, T046, T184 and T190 showing strong gains. This indicates the potential variance in the data sample.

**Table 10:** **Type-based learn to rank on test data**

**ID**	**Sys**	**P**	**R**	**F1**	**ID**	**Sys**	**P**	**R**	**F1**
T019	SVMRank	0.51	0.21	**0.29**	T047	SVMRank	0.52	0.66	**0.58**
	ListNet	0.59	0.18	0.28		ListNet	0.41	0.65	0.50
	RankNet	0.54	0.16	0.24		RankNet	0.38	0.51	0.44
	RankBoost	0.47	0.21	**0.29**		RankBoost	0.45	0.68	0.54
T020	SVMRank	0.29	0.53	0.37	T048	SVMRank	0.48	0.68	**0.56**
	ListNet	0.34	0.50	**0.41**		ListNet	0.40	0.49	0.44
	RankNet	0.34	0.48	0.40		RankNet	0.38	0.48	0.43
	RankBoost	0.29	0.55	0.38		RankBoost	0.44	0.68	0.54
T033	SVMRank	0.00	0.07	0.00	T184	SVMRank	0.52	0.62	**0.57**
	ListNet	0.00	0.27	0.00		ListNet	0.44	0.61	0.51
	RankNet	0.00	0.27	**0.01**		RankNet	0.40	0.58	0.47
	RankBoost	0.00	0.20	0.00		RankBoost	0.48	0.62	0.54
T037	SVMRank	0.37	0.50	**0.43**	T190	SVMRank	0.28	0.69	**0.40**
	ListNet	0.35	0.46	0.40		ListNet	0.28	0.65	0.39
	RankNet	0.31	0.44	0.37		RankNet	0.25	0.55	0.34
	RankBoost	0.35	0.49	0.41		RankBoost	0.28	0.68	0.39
T046	SVMRank	0.37	0.70	**0.48**	T191	SVMRank	0.27	0.54	**0.36**
	ListNet	0.37	0.70	**0.48**		ListNet	0.23	0.34	0.27
	RankNet	0.36	0.56	0.44		RankNet	0.17	0.24	0.20
	RankBoost	0.35	0.70	0.47		RankBoost	0.25	0.53	0.34

Note that the highest scoring system F1 for each semantic type is shown in bold.

## Discussion

### Examples of complications

**Short forms** Whilst we still need to conduct a detailed drill down analysis we can see from a preliminary survey that one of the most significant sources of error is the strong prevalence of undefined abbreviations in the clinical texts, e.g. ‘cp’ for C0008031: [chest pain], ‘la enlargement’ for C0344720: [left atrium enlargement], ‘n’ for C0027497: [nausea]. Without pre-processing to normalise to full forms, the degree of ambiguity in the short forms causes difficulties for the four systems which cannot be solved in the ensemble. In contrast, full forms of short forms were often found by the approaches employed.

**Lack of context** A common problem in clinical texts is known to be a lack of grammatical context. For example, a line in a record might consist only of a single noun phrase without end of line punctuation such as “Left bundle branch block” C0023211: [left bundle branch block]. Whilst this should in theory be less of a problem for algorithms that employ only local contextual patterns it, nevertheless, presents issues for sentence boundary detection, which might introduce unexpected errors. In shortened sentences, omission of the subject is often a problem, e.g. ‘relative afferent defect’ can only be fully understood in the context of the preceding sentence referring to ‘ocular discs’ and therefore achieving a normalisation on C0339663: [afferent pupillary defect].

**Complex grammatical structures and inferences** Disjoint concept mentions and inferences add an extra layer of difficulty to the task. An example including a long distance relationship as well as an inference is shown in the following sentence: ‘On motor exam, there is generally decreased bulk and tone, decreased symmetrically, there is generalised wasting …’. Firstly, an inference is required to find the anatomical entity in question, which in this example is the *muscle* indicated by ‘motor exam’ and the context provided in the sentence ‘decreased bulk and tone’ and ‘wasting’. Secondly, the inferred entity then needs to be connected with other distant text spans in the sentence such as ‘generally decreased bulk and tone’ and ‘generalised wasting’ to yield the intended annotations C0026846: [muscle wasting] and C0026827: [decreased muscle tone]. However, we note here that inference is not consistently handled in the gold standard. For example’, … the gastrointestinal service felt that an upper gastrointestinal bleed secondary to non-steroidal anti-inflammatory drugs was …’ is annotated with C0413722: [non-steroidal anti-inflammatory drugs] in the gold standard, suppressing the information that there is an adverse reaction (‘upper gastrointestinal bleed secondary to’). If a system were to use matching and local context rules, it may miss this annotation as its inference system would expect to annotate ‘secondary to non-steroidal anti-inflammatory drugs’, which, to the best of our knowledge, does not exist as an ontology concept.

**Coordination** Coordinating terms occur in a variety of forms, e.g. in comma lists or with ‘and’ and ‘or’ leading to head sharing. For example, ‘abdomen soft, non-tender, non-distended’ should give C0426663: [abdomen soft] and C0424826: [abdomen non-distended]. Whilst short forms and coordination are known issues that are handled by state-of-the-art biomedical named entity recognition pipelines, the lack of context in clinical reports and in particular the disjointed nature of some complex phenotypes has not yet been adequately considered

### Comparison with other ensemble approaches

Although there has been quite a lot published on the subject of concept normalisation and a large body of literature on named entity recognition, there is relatively little work on comparing and combining existing systems in ensemble approaches. In particular, learn-to-rank is a fairly recent technique for concept normalisation. To the best of our knowledge, it has only been applied once before by Leaman et al. [35] for diseases, a subset of the semantic types that we test here. Leaman et al. report promising results on a subset of the NCBI disease corpus and, in fact, their system came first in the ShARE/CLEF Task 1b.

Ensembles have though been used before for the recognition of clinical concepts. Kang et al. [36] for example employed dictionary and statistical pattern based techniques on the 2010 I2B2 corpus of EPRs, for term recognition (but not concept normalisation) achieving the third level of performance in the shared task. Xia et al. [37] show the effects of combining MetaMap and cTAKES for the same ShARE/CLEF data we have shown here. Their combination strategy is a simple rule-based approach that accepts all outputs from the higher precision system and then checks for conflicts in the output of the high recall system before accepting new CUIs.

One line of investigation we want to pursue in future work is to decouple the ranking of concepts from system baskets, i.e. instead of treating the rank of a whole basket of concepts as the target we provide individual concepts for each system and then learn to rank these. This would potentially allow us to better control for systems that are strong on some concepts and weaker on others.

### Limitations

All of the individual systems applied in our base study were used without customization, e.g. training or special post-processing rules. This is in contrast to the systems in the ShARE/CLEF 2013 shared task which usually employed machine learning on the labelled target domain data to detect relevant spans of text for named entities and to filter the suggested concept identifiers so that they were optimized for the detected spans. Both of these steps led to substantial improvements on the results of the uncustomized individual systems that we report here. We believe that in particular the lack of a post-processing step to filter concepts which did not directly appear in the text or were overlapping with other concepts led to substantially degraded precision than shared task participants. For example we found that our individual systems suggested many unannotated concepts related to the patient such as date of birth, gender, age and history of illness as well as generic concepts that were part of more specific ones. The best tuned system in the ShARE/CLEF 2013 Task 1 (named entity recognition and normalization to SNOMED-CT at mention level) achieved an F1 of 0.75 for named entity recognition and an accuracy score of 0.59 for harmonization using strict matching criteria. Taken together with the F1 improvement we observed in the ensemble approach, this finding reinforces the generally held view that domain tuning is a necessary step to achieving high F1, even with relatively mature concept recognition tools such as the ones we have employed.

Our choice of sentence-level concept harmonisation was motivated by a use-case where the user requires extraction of concepts from the document, e.g. for document or section classification, but does not require intra-sentential relationships between concepts, e.g. for text mining. The later would require mention-level harmonisation by the four individual systems but our previous experiments [38] have again indicated the challenge of attempting this without some form of tuning. In future work we would like to look at expanding our approach to exploit domain-adaptation methods, e.g. Latent Dirichlet Allocation (LDA), on mention-level annotation to allow direct comparison with the techniques employed in ShARE/CLEF 2013.

## Conclusions

Clinical phenotype recognition is essential for interpreting the evidence about human diseases in clinical records and the scientific literature. In this paper, we have evaluated the F1 of four off-the-shelf concept recognition systems for identifying some of the building blocks in clinical phenotypes as well as disease-related concepts. Future work will have to develop additional filters for this purpose. Our investigation of LTR techniques has clearly shown that the methods we adopted are superior to the off-the-shelf systems used separately but still fall short of Oracle-based settings indicating that further enhancements are required in either feature selection or sampling.

The tests have been run on the open gold-standard ShARE/CLEF corpus harmonised to UMLS semantic types. Findings indicate that cTAKES performs well compared to its peers but that annotation performance varies widely across semantic types, and that MetaMap with strict matching and word sense disambiguation can have superior precision. We presented an approach using several learn-to-rank methods that gave greatly improved performance across semantic types. The best ensemble - SVMRank - using the union tie-breaking strategy and the oracle tie-breaking strategies achieved the Top-1 ranking level on training data. The results on the test data were similar with both tie-breaking strategies at Top-1 ranking.

The results indicate the continued challenge of concept annotation and, in particular, the need to consider the grammatical relations within phenotype mentions. We have not yet tested the effectiveness of these approaches in an operational setting, e.g. for speed of processing or stability. We would like to extend our approach on further clinical benchmark data sets as they become available in order to understand better the relative merits of external feature sets such as FB3 and FB4. In the immediate future, we plan on continuing to improve our approach by extending the distributed feature representation employed in the meta-classifier, e.g. with LDA, and by exploring additional ways of sampling and combining system outputs.
